# Opportunities for early oral therapy for prosthetic hip and knee joint infections (PJI): clinical experience at a large health authority

**DOI:** 10.1017/ash.2025.10229

**Published:** 2025-11-20

**Authors:** Maggie Wong, Kevin Afra, Davie Wong

**Affiliations:** 1 University of British Columbia, Fraser Health Authority, Vancouver, Canada; 2 Department of Pharmacy, Royal Columbian Hospital, New Westminster, BC, Canada; 3 Division of Infectious Diseases, Department of Medicine, Surrey Memorial Hospital, Fraser Health, Surrey, Canada; 4 Division of Infectious Diseases, Department of Medicine, University of British Columbia, Vancouver, Canada; 5 Division of Infectious Diseases, Department of Medicine, Royal Columbian Hospital, New Westminster, BC, Canada

## Abstract

**Objectives:**

We described the clinical outcomes and estimated cost savings from switching patients to early oral therapy from intravenous (IV) therapy for prosthetic joint infections (PJI) based on predefined criteria.

**Methods:**

Retrospective observational study in a large health authority consisting of 12 acute care hospitals in Canada. Patient demographics, microbiological and treatment data were collected for all patients with first episode of knee or hip PJI in 2022. Treatment failure rates, allergic or adverse reactions to IV or oral treatment, and hospital readmission rates were reported for those who met criteria for early switch to oral therapy.

**Results:**

Fifty-one patients were included. Thirty-seven patients (73%) had knee PJI, with debridement, antibiotics, and implant retention being the most common procedure. Sixteen patients (31%) had IV therapy for the entire duration of treatment, and the mean duration was 44 days. Twenty-three patients (45%) could have been switched to oral therapy. In practice however, only 3 patients (6%) were switched to oral therapy by day 7 following surgical source control. Five patients (22%) had clinical and/or microbiological failure 2 years postsurgery. Four patients (17%) and 6 patients (26%) developed an allergic or adverse reaction to IV and oral therapy, respectively. Five patients (22%) developed line complications. We estimated potential cost savings of almost $70,000 Canadian dollars with early oral therapy.

**Conclusion:**

Almost half of our PJI patients could have been switched to oral therapy within 7 days post-surgical source control. This study highlights a great opportunity for antimicrobial stewardship.

## Background

The prevalence of prosthetic hip and knee joint infections (PJI) were over 2% at 10 years.^1^ With the aging population, this rate may rise which increases patients’ risk of morbidity and mortality. Prolonged duration of intravenous (IV) antimicrobial therapy was recommended by the Infectious Diseases Society of America (IDSA), which was last updated in 2013.^2^

Since then, a randomized controlled study (OVIVA—Oral vs IntraVenous Antibiotic treatment for bone and joint infections) demonstrated that oral therapy was non-inferior to IV therapy for bone and joint infections.^3,4^ A UK study at an orthopedic hospital found that 66.1% of patients could have been switched from IV to oral antimicrobial(s) following the OVIVA study protocol. They noted numerous benefits with oral therapy, such as shortening the length of hospital stay and reducing line complications.^5^ These studies highlight that patients do not need to wait for a prespecified period prior to de-escalation to oral therapy if they meet certain criteria. For the duration of therapy, recent literature has shown that it can be as short as 12 weeks for patients with a retained implant, and 4 to 6 weeks for one and two stage exchange procedures.^6,7,8,9^

In the OVIVA study, PJI comprised a small subset of bone and joint infections.^3^ Therefore, our study focused solely on PJI with the following objectives: 1) Describe the current practice of PJI treatment in our health authority; 2) Determine the proportion of patients who could have been switched to oral antimicrobial(s) from IV therapy based on predefined criteria by day seven; and 3) Describe clinical outcomes and potential cost savings in patients who met criteria for early oral switch therapy.

## Methods

### Setting

Our health authority consists of 12 acute care hospitals serving over two million people in British Columbia, Canada. We have regional infectious diseases (ID) physicians on site who can also provide phone consultation advice. Our hospitals are publicly funded. The outpatient parenteral antimicrobial therapy (OPAT) service is delivered via daily attendance at hospital clinics. Home IV programs offer self-administration of IV medications by patients or family members in their own home. The cost of equipment and IV drugs associated with the OPAT and home IV program are covered by the health authority. The cost of oral medications are paid by patients, although they can potentially be covered by public or private health insurance.

### Inclusion and exclusion criteria

This is a retrospective, observational study from January 1, 2022, to December 31, 2022, across all the sites. We included all patients > 18 years old with a first case of hip or knee PJI. A list of patients with 2 or more positive operative cultures from knees or hips was provided by the microbiology lab. A data analyst cross-referenced the list with ICD-10 codes to identify patients with prosthetic knee or hip infections. Subsequent chart reviews were done to confirm that these cases followed the definition of PJI per the IDSA guideline

Exclusion criteria were based on the OVIVA trial protocol and described as below.^4^ Patients were excluded from oral step-down within the first week of treatment if they met any of these criteria: 1. *Staphylococcus aureus* bacteremia and/or endocarditis on presentation or within the previous month; 2. Any concomitant infection requiring a prolonged course of IV therapy (eg, central nervous system infection); 3. Patient had sepsis or deemed clinically unstable by the most responsible physician(s); 4. A lack of suitable oral antibiotic choices (eg, organisms were only sensitive to IV antibiotics, drug interaction, drug allergies); 5. Clinical, histological, or microbiological evidence of mycobacterial, fungal, parasitic, or viral etiology of the infection; 6. Patient had swallowing difficulties or unable to adequately absorb oral medication(s); 7. Patient without any surgical source control; 8. Body weight > 100 kg where weight-based dosing is important for efficacy of antibiotic(s); 9. Not likely to be compliant with oral antibiotic(s) at the discretion of ID physician.

### Data collection and outcome(s) measurement

Demographics (age, sex, comorbidities), symptoms onset, type of orthopedic procedures, microbiological results from surgery, length of hospital stay, antimicrobial(s) choice and duration, and disposition were reported for all patients.

Primary and secondary outcomes were reported only for those who could be switched to oral therapy based on predefined criteria. Primary outcomes included treatment success and failure rates within 2 years postsurgery. Treatment failure was a composite of clinical and microbiological outcomes defined as 1. Formation of a draining sinus tract arising from bone or prosthesis and/or recurrence of frank pus adjacent to bone or prosthesis, and 2. Isolation of bacteria from two or more samples of peri-prosthetic tissue.

Secondary outcomes included: allergic or adverse reaction(s) related to IV and/or oral antimicrobial(s), 30- and 90-day hospital readmission rates and mortality rates after stopping treatment, and line complication(s) from IV antimicrobial(s).

An ID pharmacist and an ID fellow reviewed the first twenty percent of the cases together to ensure that there was no protocol violation. Any disagreement with outcome adjudication would be further discussed with other authors to ensure group consensus was achieved.

### Economic analysis

The cost savings for each patient were estimated by calculating the difference between the inpatient plus outpatient IV expenses and the cost of a hypothetical oral regimen that could have been used as definitive therapy. (See supplementary material for details)

### Statistics

All collected data were reported using descriptive statistics.

Since this was a retrospective study for program evaluation and quality improvement, it was exempted from ethical approval by our local health authority.

## Results

Of 58 patients screened, 51 were included. Seven patients were excluded due to not meeting the definition of PJI on chart review, had previous episodes of PJI, or non-hip or -knee PJI. Forty-nine patients (96%) had ID consultations in-person or via phone. The mean age of patients was 69 years. Type 2 diabetes and cardiovascular diseases were the most common comorbidities. Twenty-four patients (47%) had early-onset symptoms of PJI. Thirty-seven patients (73%) had knee PJI, with debridement, antibiotics, and implant retention (DAIR) being the most common surgical procedures. Sixty-seven percent of identified pathogens were Gram-positive bacteria; the remaining were polymicrobial (18%) or Gram-negative bacteria (12%). Sixteen patients (31%) had IV therapy for the entire duration of treatment, and the mean duration was 44 days. Thirty-one patients (61%) were transitioned to oral therapy after at least 2 weeks of IV treatment. The mean duration of oral therapy was 128 days, excluding 9 patients on indefinite suppressive therapy. Patients’ demographics, microbiological results, treatment choices and duration are summarized in Table 1.

**Table 1.** Patient demographics, microbiological results and treatment for PJI

Patient demographics and culture results from surgical samples (N = 51)	
Age (years)	Mean: 69.3 (ranged from 44 to 86) Median: 69
Sex (%)	Male: 28 (55) Female: 23 (45)
Comorbidities (%)	Type 2 diabetes: 13 (25) Cardiovascular disease: 13 (25) Chronic obstructive pulmonary disease: 10 (20) Immunosuppression: 5 (10) Cancer: 5 (10) Peripheral vascular disease: 4 (8) Chronic kidney disease: 4 (8) Liver disease: 1 (2)
Type of infections (%)	Knee: 37 (73) Hip: 14 (27)
Onset of symptoms	Early (< 3 mo post last procedure): 24 (47) Delayed (3–12 mo post last procedure): 11 (22) Late (> 12 mo post last procedure): 16 (31)
Type of orthopedic procedures (%)	Debridement, antibiotics, and implant retention (DAIR): 37 (73) Two-stage exchange: 13 (25) Girdlestone (resection arthroplasty): 1 (2) One-stage: 0
Length of hospital stay (days)	Mean: 28 (range from 4 to 135) Median: 12
Microbiology from surgical samples (%)	Methicillin susceptible *staphylococcus aureus* (MSSA): 16 (31) Methicillin resistant *staphylococcus aureus* (MRSA): 5 (10) Streptococcus species: 11 (22) Coagulase negative staphylococci: 1 (2) Enterococcus species: 1 (2) Enterobacterales: 5 (10) *Pseudomonas aeruginosa*: 1 (2) Culture-negative: 1 (2) (note: patient was already on antibiotics prior to sample) Polymicrobial: 9 (18) Fungi: 1 (2)
**Treatment and duration characteristics** N = 51 (unless specified)	
Definitive intravenous (IV) antimicrobial(s) (%) *n* = 50	Cefazolin: 16 (32) Ceftriaxone:13 (26) Daptomycin: 9 (18) Carbapenems: 6 (12) Penicillin: 2 (4) Piperacillin-tazobactam: 2 (4) Ampicillin: 1 (2) Fluconazole: 1 (2) Note: 1 patient was switched to po antibiotic promptly upon culture result and not on definitive IV therapy
Duration of IV treatment (days)	Mean: 44.2 Median: 42
Definitive oral antimicrobial(s) that patient was prescribed (%) *n* = 34 Note: patient can be on more than 1 antimicrobial	Doxycycline: 13 Amoxicillin: 9 Levofloxacin: 5 Cephalosporins (cephalexin, cefadroxil): 4 Ciprofloxacin: 3 Trimethoprim/sulfamethoxazole: 3 Clindamycin: 2
Duration of oral treatment (days)	Mean: 128 Median:150
Patient disposition (%)	Home IV: 24 (47) Finished oral therapy at home: 10 (20) Outpatient parenteral antimicrobial therapy: 5 (10) Finished IV therapy at hospital: 12 (23)
Concurrent use of rifampin (%)	16 (31)

Based on predefined criteria, 23 patients (45%) could have been switched to oral therapy. In practice however, only 3 patients (6%) were switched to oral therapy by day 7 following surgical source control. Primary and secondary outcomes were based on these patients. Five patients (22%) had clinical and/or microbiological failure 2 years postsurgery. Four patients (17%) and 6 patients (26%) developed an allergic or adverse reaction to IV and oral therapy, respectively. Five patients (22%) developed line complications. Primary and secondary outcomes are summarized in Table 2.

**Table 2.** Primary and secondary outcomes

Primary and secondary outcomes (*n* = 23)	
Reasons for exclusion from oral switch by day 7 (number of patients)	No suitable oral antibiotic (eg, no susceptible oral agent, drug interaction, drug allergy): 9 Staphylococcus bacteremia and/or other concurrent infection requiring prolonged therapy: 8 Clinically unstable or lack of source control: 4 Weight>100 kilograms where weight-based dosing is important for efficacy: 2 Excluded pathogen (Fungal, Mycobacterial, Parasitic, or viral culprit organism): 1 Unable to swallow or absorb oral medication: 1 Low likelihood of adherence to oral medication: 1 Already on chronic suppressive oral therapy: 1
Clinical and microbiological failure at 2 years (%)	5 (22)
Adverse reactions to IV therapy	4 (17%) – 3 cases related to itchiness or rash, 1 case related to neutropenia potentially from ceftriaxone
Adverse reactions to oral therapy	6 (26%); 4 (17%) required a change in therapy or stopping antibiotic(s) – see below: Rifampin—nausea, decreased appetite (resolved with stopping therapy and continued with doxycycline alone) Doxycycline—nausea/vomiting (resolved with switching back to cefazolin) Levofloxacin—shoulder tendinopathy (resolved with stopping) Amoxicillin—diffuse rash (resolved with switching to cefuroxime) Cefadroxil—diarrhea (able to continue with therapy) Ciprofloxacin—diarrhea (able to continue with therapy)
Documented issues with adherence	None documented in patients’ charts
Readmission to hospital (%)	30-day readmission: none 90-day readmission: 2(9)
30-day mortality	None
Peripheral inserted central catheter (PICC) related issues (%)	5 (22) 4 cases (rash/drainage/irritation at PICC site) 1 case (blocked PICC)

The overall estimated potential cost saving of early oral switch therapy was $69,757 CAD (Table 3).

**Table 3.** Estimated total cost savings

Estimated Total Cost Savings (*n* = 51)		
Inpatient	Actual Cost	Potential Cost
IV antibiotics	$11,072.58	
Oral antibiotics		$112.8
Outpatient		
IV antibiotics	$7,423.80	
Oral antibiotics		$3,039.21
PICC line	$6,000	
IV supplies, visit and service fees	$48,412.38	
Total	$72,908.76	$3,152.01
Savings	$69,756.75	

## Discussion

This study demonstrates that 45% of included patients with PJI could have been switched to oral therapy within the first week postsurgery using OVIVA study protocol. On average, these patients were not switched to oral therapy until day 31 under the current model. Similar to a study on implementation of OVIVA’s criteria in the OPAT setting, we found that the main reason for inability to switch to oral therapy was a lack of a suitable agent due to microbiological or patient factors.^10^ However, for those who met the predefined criteria (17 out of 23 patients), the ID physicians did not provide any rationale for delayed oral switch in the patients’ charts.

Therefore, this study presents a great opportunity for quality improvement in terms of antimicrobial stewardship and resource allocation. For example, some of our patients could have been switched from ceftriaxone to narrower spectrum beta-lactams such as high-dose amoxicillin once susceptibilities were known. By switching patients to oral therapy, we estimated a potential cost savings of almost $70,000 CAD. Since 57% of the patients were enrolled in a home IV program or required daily IV administration at a hospital clinic to complete their IV regimen, early oral switch might decrease utilization of some of these resources, freeing them up for other patients. Even though 6 patients developed an adverse or allergic reaction to oral therapy, only 1 patient was transitioned back to IV therapy even when other oral treatment options were available. As for IV therapy, 5 patients developed some form of line complication, ranging from rash or drainage at the IV site to full blockage of the line, which required medical attention. The use of oral therapy avoids any IV-line associated issues. For 5 out of 23 patients (22%) who had treatment failure, the primary reason was related to delayed or inadequate source control. A summary of these cases was provided in supplementary material. Compared to an OVIVA implementation study by Azamgarhi et al., 73% of our patients had DAIR versus 20% in their cohort; majority of their patients had implant removal.^5^ This could potentially explain our higher failure rates as opposed to the choice or route of antibiotic(s).

The estimated total days of IV therapy that could have been averted were 684 days, assuming that all our patients who met predefined criteria could have been switched to oral therapy on day 7. Additionally, there is no evidence-based minimum duration of IV lead in before converting to oral treatment. Day 7 was chosen for practical reasons as microbiology results in our health authority are often finalized by this time. Some randomized controlled trials have a shorter IV lead in ranging from 0 – 4 days for the treatment of bone and joint infections.^11^

According to a survey by Cortés-Penfield et al., there were significant variations in the way US ID physicians used oral antibiotic(s) for osteoarticular infections. Only 31% of the respondents switched patients from IV to oral antibiotics within the first 2 weeks of treatment as definitive therapy.^12^ To encourage our ID physicians to adopt an early oral switch strategy, we recently developed a standardized guideline with suggested oral regimens and doses for common pathogens in PJI. This information is easily accessible to clinicians on our hospital website and smart phone applications. The PJI guideline was also approved by our local orthopedic surgeons. One future direction is to collect data following publication of this guideline to measure compliance with our recommendations.

Some limitations of this study include: 1. We acknowledged that our sample size (*n* = 51) was small since we excluded patients with culture-negative PJI. This was due to our ID physicians expressing concerns about switching patients to oral therapy without positive culture results for guidance. This issue was also identified by Lam et al as an area of uncertainty.^13^ With only 23 patients who could have been switched to oral therapy within the first 7 days, it limited our ability to draw robust conclusions about clinical outcomes of early switch in this population. 2. We were not able to determine whether early switch to oral therapy facilitated earlier hospital discharge due to the retrospective nature of the study. 3. Results may not be generalizable to centers with different orthopedic practice. For example, most of our patients had DAIR where the duration of therapy is longer compared to two stage exchange procedures, and none had one stage exchange. 4. Our cost calculation likely under-estimated the true savings since we were unable to account for indirect costs (see supplementary material).

In conclusion, our study demonstrated that almost half of the patients were candidates for early oral switch therapy in their treatment course of PJI. Our real-world data adds to the existing literature challenging the dogma that IV is better than oral antibiotics for the treatment of PJI.

## Supporting information

10.1017/ash.2025.10229.sm001 Wong et al. supplementary material Wong et al. supplementary material

## Supplementary material

The supplementary material for this article can be found at https://doi.org/10.1017/ash.2025.10229.
