# Supplementary material for: Opportunities for early oral therapy for prosthetic hip and knee joint infections (PJI): clinical experience at a large health authority
Source: Antimicrob Steward Healthc Epidemiol. 2025 Nov 20;5(1):e311. doi: 10.1017/ash.2025.10229 (PMC12645242; doi:10.1017/ash.2025.10229)
Supplement: Wong et al. supplementary material [file S2732494X25102295sup001.docx]

**Supplemental material**

Table 1: Cases with treatment failure (n=5)

|  | **Description of the patient, surgery received and microbiological culture** | **Treatment (intravenous or oral) received** | **Outcome** |
| --- | --- | --- | --- |
| Case 1 | 44-year-old female with delayed methicillin-susceptible *S. aureus* hip infection. She had debridement, antibiotics and implant retention procedure (DAIR). | Cefazolin for 1 week followed by doxycycline for 3 months | Required two-stage exchange surgery after 1.5 year from DAIR |
| Case 2 | 65-year-old male with late methicillin-susceptible *S. aureus* hip infection. He was treated with 2-stage exchange. | Cefazolin for 4 weeks followed by doxycycline x 2 weeks, and then cephalexin | Developed recurrent hip infection which required subsequent operation |
| Case 3 | 67-year-old female with early hip infection growing vancomycin resistant *enterococcus faecium* and *staphylococcus epidermidis*. Patient had irrigation and debridement without hardware exchange. | Daptomycin for 6 weeks followed by doxycycline. | Patient developed recurrent infection, and infectious diseases physician indicated concern of no hardware exchange from the initial surgery |
| Case 4 | 71-year-old female with early hip infection. She had DAIR. Culture grew *pseudomonas aeruginosa*. | Piperacillin-tazobactam for 8 weeks | Required two-stage exchange |
| Case 5 | 60-year-old male with early knee infection treated with DAIR. Blood culture positive for *E. coli*. | On ceftriaxone for 6 weeks, followed by oral step down to levofloxacin | Required two-stage procedure as patient developed new trauma to the same knee |

**Economic Analysis**

We included the cost of inpatient and outpatient antibiotics, PICC line, home IV supplies (ie. gravity pole, CADD pump, syringes) and service fees in the economic analysis. We were unable to determine indirect costs such as length of hospital stay, potential loss of income from inability to work, IV-line related complications, medication adverse effects, travel expenses or treatment failures so these were not factored into the calculation. One infectious disease physician who is familiar with the prescribing habits for PJI in the health authority determined the hypothetical oral regimen for each patient. In our health authority, the treatment standard for PJI is typically up to 6 weeks of initial IV antibiotic from surgical source control, followed by oral therapy if needed to complete a total duration of 3-6 months depending on the location of infection (hip vs knee) and type of procedure performed (6 weeks for 2 stage exchange, 3 months for hip and 6 months for knee treated DAIR).

The total expenses of the IV regimen included the cost of IV antibiotic, PICC line, home IV supplies and service fees. For the hypothetical oral regimen, only the drug costs and dispensing fees were factored into the total expenditure. The cost savings was calculated by taking the difference between the cost of the prescribed IV regimen and the cost of the hypothetical oral regimen from day 7 to the end of IV therapy, which is usually up to 6 weeks from date of surgery.
